# Expression analysis and biological regulation of silencing regulatory protein 6 (SIRT6) in cutaneous squamous cell carcinoma^☆^

**DOI:** 10.1016/j.abd.2023.08.010

**Published:** 2024-03-27

**Authors:** Sai Chen, Hongxia Chen, Xu Wang, Dongmei Zhang, Li Zhang, Jiawei Cheng, Qi Zhang, Zhixiang Hua, Xu Miao, Jian Shi

**Affiliations:** a Department of Dermatology, Affiliated Hospital 2 of Nantong University, People’s Republic of China; b Jiangsu Provincial Medical Key Discipline (Laboratory) Cultivation Unit of Immunology, Nantong First People’s Hospital, People’s Republic of China; c Medical Research Center, Affiliated Hospital 2 of Nantong University, People’s Republic of China

**Keywords:** Biomarkers, Carcinoma, squamous cell, Histology, Sirtuin-6

## Abstract

**Background:**

Cutaneous squamous cell carcinoma (CSCC) is one of the most common types of skin cancer worldwide. Therefore, the identification of biomarkers associated with CSCC progression could aid in the early detection of high-risk squamous cell carcinoma and the development of novel therapeutic strategies.

**Objective:**

This study aimed to investigate the expression patterns of silent mating type Information Regulation 2 homolog 6 (SIRT6) in CSCC and its clinical significance.

**Methods:**

The protein expression level of SIRT6 in tissues was detected by immunohistochemistry, and the correlation between SIRT6 expression and clinicopathological parameters in CSCC patients was analyzed. The relative expression of SIRT6 in CSCC cell lineage and tissue specimens was determined by western blotting and PCR. The effect of SIRT6 silencing on cell proliferation was evaluated using cell counting kit 8. Wound healing, transwell method, and flow cytometry were used to investigate the migration, invasion, and cell cycle distribution/apoptosis of CSCC cells after SIRT6 silencing, respectively. Western blot was used to detect the expression of EMT (Epithelial-Mesenchymal Transition), cycle, apoptosis, and other related proteins.

**Results:**

The high expression of SIRT6 was correlated with the location of cancer tissue and Broder staging in CSCC patients. Knockdown of SIRT6 inhibited the proliferation, migration, invasion and EMT of CSCC cells, and promoted their apoptosis, with cells blocked in G1 phase.

**Study limitations:**

No animal experiments were conducted to further verify the results.

**Conclusion:**

Decreased expression of SIRT6 can inhibit the occurrence and development of CSCC.

## Introduction

Cutaneous squamous cell carcinoma (CSCC) is emerging as the second most common non-melanoma skin cancer, with an increasing incidence ranging from 20% to 30% of all cutaneous malignancy.^1^ The progression of CSCC is determined by a combination of genetic susceptibility and environmental risk factors, including exposure to ultraviolet radiation, carcinogens and immunosuppressive drugs.^2^ Despite the relatively low risk of metastasis and favorable long-term survival after treatment, the incidence of CSCC has increased proportionately in recent years, resulting in significant long-term morbidity, mortality, and economic burden for patients.^3^, ^4^, ^5^ Therefore, a deeper understanding of the pathogenesis of CSCC is crucial for improved diagnosis and treatment efficacy.^6^

Sirtuins, which belong to the histone deacetylase family, are involved in various cellular functions such as aging, stress response, metabolism, and energy homeostasis.^7^, ^8^, ^9^ Mammalian sirtuins consist of seven family members (SIRT1-7), which exhibit different subcellular localization, catalytic activities, and biological functions.^10^ SIRT6, primarily located in the nucleus, functions as a highly conserved NAD+-dependent deacetylase and ADP-ribosyltransferase.^11^, ^12^ SIRT6 has been implicated in diabetes, cardiovascular disease, aging, and cancer.^13^, ^14^, ^15^, ^16^ Emerging evidence suggests a strong link between SIRT6 and the initiation and progression of multiple myeloma (MM) and several tumor types.^17^, ^18^ Its dual role of either suppressing or promoting cancer has been observed in different cancer types, potentially influenced by signaling pathways and tissue-specific contexts.^19^, ^20^ However, the precise impact of SIRT6 on CSCC remains largely elusive. The authors hypothesize that SIRT6 plays a pivotal role in the pathogenesis and progression of CSCC, prompting us to evaluate its expression patterns, functional mechanisms, and potential clinical implications.

## Materials and methods

### Materials

#### Reagents

The following reagents were used: A431 human cutaneous squamous cell carcinoma cell line (Obtained from Zhongqiao Xinzhou, Shanghai) and SCL-1 cell line (Enzyme Research, Shanghai); The normal immortalized human keratinocyte line HaCaT (Shanghai Fuheng Biotechnology Co., Ltd.);skim milk, chloroform, and isopropanol (purchased from Shanghai Shenggong Co., Ltd.); primary antibodies against SIRT6 and β-tubulin, secondary antibodies against mouse immunoglobulin IgG (purchased from Abcam, USA); mouse anti-human N-cadherin monoclonal antibody and mouse anti-human Vimentin monoclonal antibody (Proteintech, Wuhan); fetal bovine serum and high-glucose DMEM medium (purchased from Gibco, USA); BCA analysis kit, crystal violet, and Lipofectamine 2000 (purchased from Biyuntian Biotechnology Co., Ltd.); Polyvinylidene Fluoride (PVDF) membrane (purchased from Millipore, USA); Trizol reagent, RNA prep pure Tissue Kit, PrimeScript RT synthesis kit, and SYBR Green qRT-PCR Master Mix kit (purchased from Thermo, USA); CCK-8 assay kit (Proteintech, Wuhan); matrix gel and 8-µm transwell chambers (Corning, USA); all primers were synthesized by Shanghai Shenggong Co., Ltd.

#### Tissue samples

Tissue blocks from 70 patients with CSCC, 25 patients with BD (Bowen Disease), and 60 patients with AK (Actinic Keratosis) were collected from January 2017 to December 2022. In addition, 20 normal skin plastic surgery tissue pieces were collected as normal controls. Fresh CSCC and NT samples were collected, 20 cases each. In addition, none of the patients in this study received any treatment before surgery, including chemotherapy or radiation. All patients participating in the study obtained informed consent, and this study was approved by the Ethics Committee of this hospital (ethics approval number: 2021KT085).

### Methods

#### Immunohistochemistry for protein expression analysis

CSCC tissue samples were deparaffinized and treated with hydrogen peroxide for 5 minutes to inhibit endogenous peroxidase activity. Goat serum blocking solution was added and antigen retrieval was performed with citrate solution. CSCC tissue samples were incubated with mouse anti-SIRT6 antibody (1:200) overnight at 4 °C. After two washes with PBS for 3 minutes each, the samples were incubated with HRP-conjugated goat anti-mouse IgG (1:2000) for 1.5 hours at room temperature. After two washes with PBS for 3-minutes each, the primary antibody was detected using a Diaminobenzidine (DAB) substrate kit. Immunohistochemical staining images were observed under a microscope and several fields were randomly selected for observation at high magnification. The immunohistochemical staining results were interpreted using a commonly used semi-quantitative scoring method^21^ (Table 1).

**Table 1:** SIRT6 staining results evaluated using a 12-point semi-quantitative scoring method.

Evaluation indication	Grading		Score
Positive cell count	0%～25%		1
	26%～50%		2
	51%～75%		3
	76%～100%		4
Staining intensity	No coloring		0
	Faint yellow		1
	Claybank		2
	Tan		3
Expression intensity (Positive cell count × Dyeing strength)	Low expression	Negative (−)	0～3
		Weak Positive (+)	4～6
	High expression	Positive (++)	7～9
		Strong Positive (+++)	10～12

#### Cell culture

The cell lines used in this study included the human skin squamous cell cancer cell line (A431, SCL-1) and the normal immortalized human keratinocyte line HaCaT. The medium used for A431, SCL-1 and HaCaT cells was DMEM high-glucose medium. All cells were cultured at 37 ° with 5% CO and 10% fetal bovine serum in the medium.

#### Western blot

Cell lysis was performed using RIPA buffer and protein was extracted on ice. Protein concentration was determined using a BCA assay kit. Protein samples from each group were loaded onto a 12.5% gel and electrophoresed at 80 V for 30 minutes, followed by 110 V for 1 hour. PVDF membrane transfer was performed at 100 V in an ice bath for 1.5 hours. The membrane was then blocked with 5% skim milk for 2 hours, followed by incubation with primary antibody at 4 °C overnight and incubation with secondary antibody for 2 hours. The secondary antibodies used were goat anti-mouse HRP (1:1000 dilution) and goat anti-rabbit HRP (1:1000 dilution). Finally, images were captured and stored using a Bio-Rad chemiluminescence imaging system.

#### Real-time Reverse Transcription Polymerase Chain Reaction (qRT-PCR)

Cell pellets were collected and treated with Trizol reagent for lysis, followed by chloroform/isopropanol extraction to separate and precipitate RNA. The total RNA was further extracted by ethanol precipitation. Reverse transcription of 1 μg RNA into cDNA was performed using the PrimeScript RT synthesis kit. qRT-PCR was performed using the SYBR Green qRT-PCR Master Mix kit, with U6 used as an internal reference. The primer sequences are listed in Table 2.

**Table 2:** Primer sequences used for real-time fluorescence quantitative PCR.

Gene	Primer sequence
SIRT6	F:5′- TGGCAGTCTTCCAGTGTGGTGT-3′
	R:5′- CGCTCTCAAAGGTGGTGTCGAA-3′
U6	F:5′- CTCGCTTCGGCAGCACA -3′
	R: 5′- AACGCTTCACGAATTTGCGT-3′

#### Cell infection and screening

Lentiviral shSIRT6 and control shNC were provided by Youxi Weinan Biotechnology Company (Sanming, China). The lentiviruses were packaged using a triple plasmid system. The shRNA was inserted into the Doxycycline (DOX)-inducible gene expression vector Tet-pLKO.1-puro. After transfection for 36 hours, the lentiviruses were harvested. A431 cells and SCL-1 cells were infected with the lentiviruses for 24 hours and stable transduced cell lines were selected using puromycin (0.8 μg/mL for A431 cells, 0.4 μg/mL for SCL-1 cells). Gene expression was induced with 1 μg/mL DOX and empty vector-transfected cells served as Normal Control (NC) cells. Cells were divided into four groups: NC group, NC + DOX group, shSIRT6 group, and shSIRT6 + DOX group.

#### Cell Counting Kit-8 (CCK-8) assay for cell proliferation

Cells were seeded at 2,000 cells/100 μL in a 96-well plate with 4 replicate wells per group. The plate was incubated at 37 °C with 5% CO_2_ and saturated humidity until the cells reached 80% confluence. Each group of cells was treated differently and incubated for a further 24 hours under the same conditions. The original culture medium in each well was discarded and 100 µL of culture medium containing 10% CCK-8 was added. The plate was incubated in a cell incubator for 1 hour and the absorbance at 450 nm was measured using a microplate reader. Absorbance values (OD) were measured at 12, 24, 48 and 72 hours to assess cell proliferation.

#### Transwell assay

Transwell coated with Matrigel was used to perform cell invasion and migration assays for each group. Fifty microliters of Matrigel (2 mg/mL) were added to the upper chamber of the transwell and incubated at 37 °C for 30 minutes. Then 4 × 10^3^ cells were seeded in a serum-free DMEM medium. The lower chamber was filled with 600 μL of medium containing 10% fetal bovine serum as a chemoattractant. After incubation for 24 hours, non-invading cells were removed with a cotton swab. Invaded cells on the lower membrane were fixed with 4% paraformaldehyde for 30 minutes at room temperature and stained with 0.1% crystal violet for 20 minutes. The invaded and migrated cells were photographed and counted under a microscope (Carl Zeiss, Germany). Three random fields were selected for cell counting and the average was calculated.

#### Cell scratch assay

Log-phase cells were harvested, centrifuged, and resuspended in a culture medium. Cells were counted and each group of cells was seeded in a 6-well plate (6.0 × 10^5^ cells/well) for culture. When cell confluence reached approximately 95% the next day, a scratch was made in the center of each well using a 200 μL pipette tip. After washing with PBS, a fresh culture medium was added. The 0 -h image was taken using an inverted light microscope and the cells were further incubated. Images were taken at 24 and 48 hours. The healing rate was calculated using ImageJ software.

#### Flow cytometric analysis of cell apoptosis using annexin V/propidium iodide (PI) double staining

The culture supernatant was collected from each group of cells and the cells were washed with PBS, trypsinized and collected. The cells were double stained using the Annexin V-FITC Apoptosis Detection Kit. Staining was performed in a dark chamber at room temperature for 10 minutes. Flow cytometry was used for quantitative analysis and the apoptosis rate was calculated.

#### Cell cycle analysis

Human CSCC cell lines A431 and SCL-1 were maintained in a DMEM culture medium supplemented with 5% FBS in a 10 cm culture dish. After treatment under the specified conditions, the cells were collected and suspended in PBS. They were fixed in 70% cold ethanol overnight at 4 °C. The cells were then incubated with 50 μg/mL Propidium Iodide (PI) and 100 μg/mL RNase A (1:1) for 20 minutes at room temperature in the dark. Finally, the staining signal of CSCC cells was evaluated using the FACSCalibur™ flow cytometer (BD Biosciences, Franklin Lakes, New Jersey, USA), and the distribution of cell cycle percentages was analyzed using Cell Quest software version 3.3 (BD Biosciences) according to the manufacturer's instructions.

#### Statistical analysis

Statistical analysis was performed using SPSS 23.0 software. Quantitative data were expressed as mean ± standard deviation. The chi-Squared test was used to compare categorical data, and Analysis of Variance (ANOVA) was used to compare positive rates between multiple groups. Independent samples *t*-tests were used for pairwise comparisons. A p-value <0.05 was considered statistically significant.

## Results

### Elevated expression of SIRT6 in AK, BD, and CSCC tissues compared to normal skin tissue

**Figure 1:**
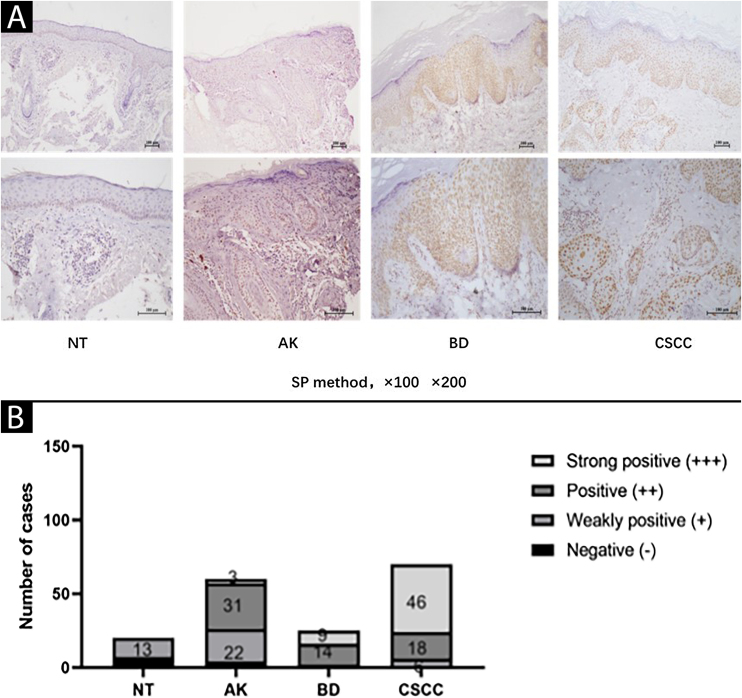
Expression of SIRT6 in NT (Normal skin Tissue), AK (Actinic Keratosis), BD (Bowen Disease), and CSCC (Cutaneous Squamous Cell Carcinoma) tissues. (A) Representative images (magnified by ×100 × 200) of SIRT6 immunohistochemical staining in NT, AK, BD, and CSCC tissues using the SP method. (B) Bar graph illustrating the intensity of SIRT6 expression: n(NT) = 20-cases (0-cases of strong positive, 0-cases of positive, 13-cases of weak positive, and 7-cases of negative); n(AK) = 60-cases (3-cases of strong positive, 31-cases of positive, 22-cases of weak positive, and 4-cases of negative); n(BD) = 25 cases (9-cases of strong positive, 14-cases of positive, 2-cases of weak positive, and 0-cases of negative); n(CSCC) = 70-cases (46-cases of strong positive, 18-cases of positive, 6-cases of weak positive, and 0-cases of negative). Note: The full name of SP method is streptomycin peroxidase coupling method.

The expression of SIRT6 in different tissues was examined using immunohistochemical staining. The results revealed that SIRT6 protein expression was predominantly observed in the cell nucleus, exhibiting a range of hues from pale yellow to brownish-brown staining, while no significant expression was observed in the cytoplasm (Fig. 1A).

A quantitative assessment of the expression levels was conducted using a semi-quantitative scoring system. The results demonstrated the following distribution in the examined tissues: among 20 cases of Normal Skin Tissue (NT), there were no cases of strong positive, positive, weak positive, or negative SIRT6 expression; for 60 cases of AK tissue, the distribution was 3-cases, 31-cases, 22-cases, and 4-cases, respectively; in 25 cases of BD tissue, the distribution was 9-cases, 14-cases, 2-cases, and 0-cases, respectively; and among 70-cases of CSCC tissue, there were 46-cases, 18-cases, 6-cases, and 0-cases, respectively (Fig. 1B). The high expression rates of SIRT6 in NT, AK, BD, and CSCC tissues were 0%, 56.67%, 92.00%, and 91.43%, respectively (Table 3). Pairwise comparisons revealed statistically significant higher expression of SIRT6 in AK, BD, and CSCC tissues compared to NT tissues (p < 0.05), while there was no statistically significant difference in SIRT6 expression between BD and CSCC tissues.

**Table 3:** Differences in expression of SIRT6 in NT, AK, BD, and CSCC tissues.

Group	Expression of SIRT6		Total	High expression rate
	Low expression (-～+)	High expression (++～+++)		
NT	20	0	20	0
AK	26	34	60	56.67%
BD	2	23	25	92.00%
CSCC	6	64	70	91.43%

NT, Normal skin Tissue; AK, Actinic Keratosis; BD, Bowen Disease; CSCC, Cutaneous Squamous Cell Carcinoma.

### Correlation between SIRT6 expression and clinical pathological parameters in CSCC patients

Analysis of the correlation between SIRT6 expression and clinical pathological parameters in CSCC patients revealed that high expression of SIRT6 was associated with cancerous tissue located in sun-exposed areas (p < 0.05) and higher Broders' grade (p < 0.05). However, no significant correlations were observed between SIRT6 expression and clinical pathological data such as gender, age, disease duration, depth of invasion, and tumor diameter (p > 0.05). Please refer to Table 4 for details.

**Table 4:** Relationship between the expression of SIRT6 and clinical characteristics of CSCC patients.

Clinical features		Number of cases	Low expression (-～+)	High expression (++～+++)	P-value
Gender	Male	26	2	24	0.433
	Female	44	4	40	
Age	≤70	13	1	12	0.207
	>70	57	3	52	
Position	Exposure	59	1	58	0.032*
	Non-exposure	11	3	8	
Course of disease	<2-years	39	3	36	0.405
	≥2-years	31	1	30	
Invasion depth	≤3-mm	52	2	50	0.128
	>3-mm	18	2	16	
Diameter	≤2-cm	49	3	46	0.316
	>2-cm	21	1	20	
Broders classification	Ⅰ‒Ⅱ	38	4	34	0.012*
	Ⅲ‒Ⅳ	32	0	32	

### High expression of SIRT6 in CSCC cells and tissues and suppression of CSCC cell proliferation by SIRT6 knockdown

**Figure 2:**
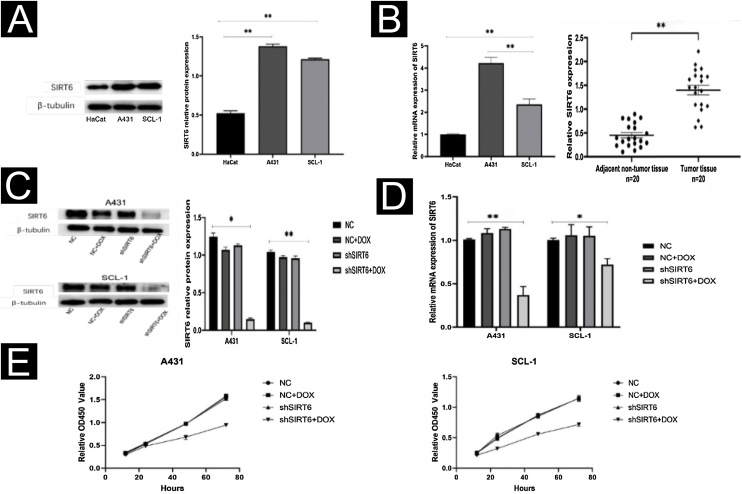
SIRT6 knockdown inhibited CSCC cell proliferation. (A) Western blot analysis of SIRT6 protein expression in A431, SCL-1, and HaCaT cells. (B) qRT-PCR analysis of SIRT6 mRNA expression in A431, SCL-1, and HaCaT cells, as well as in 20-cases of CSCC and adjacent normal tissues. (C‒D) Validation of SIRT6 protein and mRNA knockdown effects in A431 and SCL-1 cells. (E) CCK-8 assay of CSCC cell proliferation, measuring absorbance at 450 nm at 12 h, 24 h, 48 h, and 72 h; *p < 0.05, **p < 0.01.

By WB and qRT-PCR analysis, it was observed that the expression level of SIRT6 protein was higher in CSCC cell lines (A431, SCL-1) compared to immortalized human keratinocytes (HaCaT) (Fig. 2A). Similarly, the mRNA expression level of SIRT6 was higher in A431 and SCL-1 cells than in HaCaT cells. qRT-PCR analysis of 20 pairs of CSCC and adjacent normal tissues confirmed the high expression of SIRT6 in CSCC tissues (Fig. 2B), consistent with the immunohistochemistry results. To further investigate the functional role of SIRT6 in CSCC, CSCC cell lines with SIRT6 gene knockdown were established by lentiviral transduction. Negative control (NC), NC + DOX group, and shSIRT6 control group were included as negative controls. The efficiency of SIRT6 knockdown in A431 and SCL-1 cells was validated by Western blot analysis (Fig. 2C) and qRT-PCR (Fig. 2D), demonstrating downregulation of SIRT6 protein and mRNA expression in the shSIRT6 group compared to the control group. Subsequent experiments were performed using the knockdown cell lines and control groups. CCK-8 assay results showed that SIRT6 knockdown significantly inhibited the proliferation of A431 and SCL-1 cells (Fig. 2E).

### Inhibition of CSCC cell migration and invasion by SIRT6 knockdown

**Figure 3:**
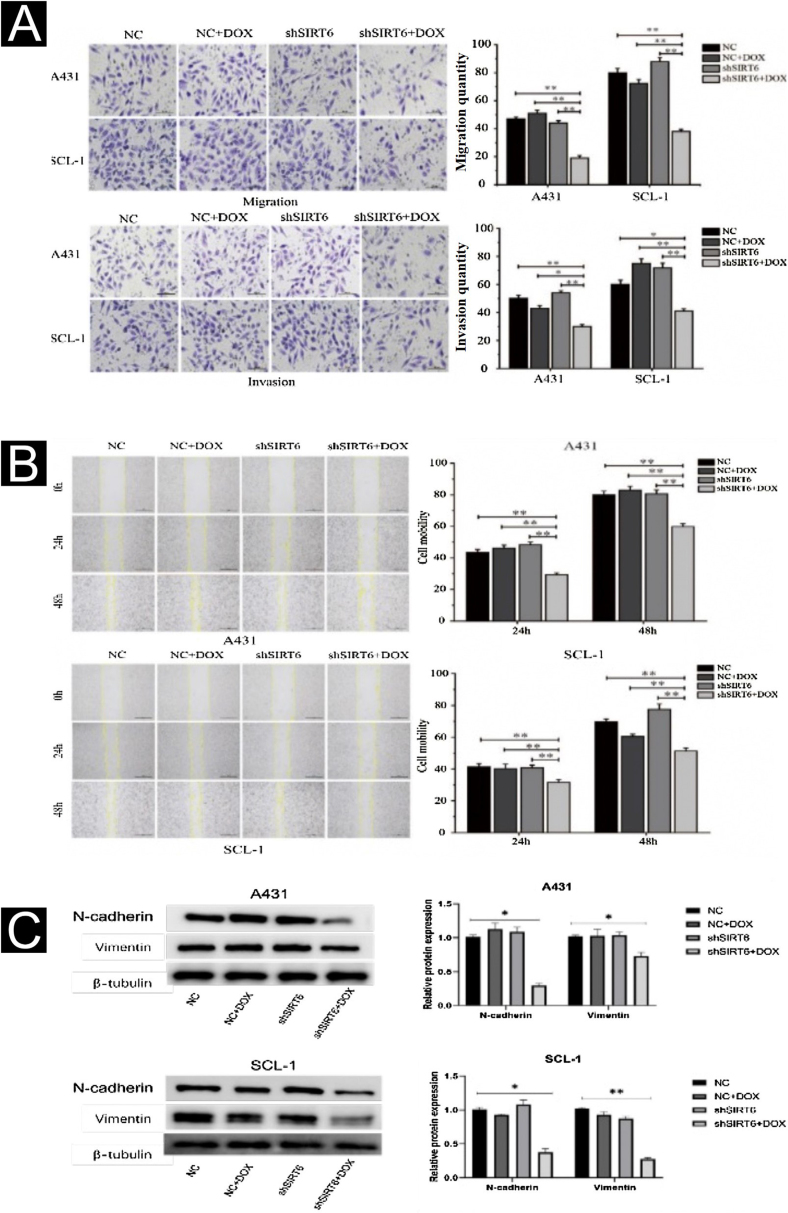
Inhibition of CSCC Cell Migration and Invasion by SIRT6 knockdown. (A) Transwell assay results and statistical analysis representing the effects of SIRT6 on cell migration and invasion in A431 and SCL-1 cells. Comparison of the number of migrated and invaded cells in the SIRT6 + DOX, NC, NC + DOX groups, and shSIRT6 control group. (B) Scratch assay results and statistical analysis demonstrating the effects of SIRT6 on cell migration rate in A431 and SCL-1 cells. Comparison of the migration rate at 24 h and 48 h in the SIRT6 + DOX, NC, NC + DOX groups, and shSIRT6 group. (C) Western blot analysis results and statistical analysis depicting the effects of SIRT6 on the expression of Epithelial-Mesenchymal Transition (EMT) markers in CSCC cells. Comparison of the expression levels of the mesenchymal markers N-cadherin and vimentin in A431 and SCL-1 cells. Note: *p < 0.05, **p < 0.01.

The effects of SIRT6 on CSCC cell migration and invasion were further investigated using the Transwell assay and the scratch assay. The results showed that compared to the control groups (NC, NC + DOX and shSIRT6 control), the shSIRT6 + DOX knockdown group exhibited reduced cell migration and invasion (Fig. 3A) and reduced wound healing rate (Fig. 3B) in A431 and SCL-1 cells. These results indicate that SIRT6 knockdown significantly inhibits the migration and invasion capabilities of CSCC cells. Western blot analysis was performed to detect the expression of epithelial-Mesenchymal Transition (EMT) markers in CSCC cells. Compared to the control groups (NC, NC + DOX and shSIRT6 control), the shSIRT6 + DOX group of A431 and SCL-1 cells showed downregulated expression of the mesenchymal markers N-cadherin and vimentin (Fig. 3C). These results indicate that SIRT6 knockdown promotes the expression of epithelial-mesenchymal transition markers N-cadherin and vimentin in CSCC cells, suggesting that the inhibition of CSCC cell migration and invasion by SIRT6 knockdown may be associated with the suppression of the EMT process.

### SIRT6 knockdown enhances apoptosis and induces cell cycle arrest at the G0/G1 phase in CSCC cells

**Figure 4:**
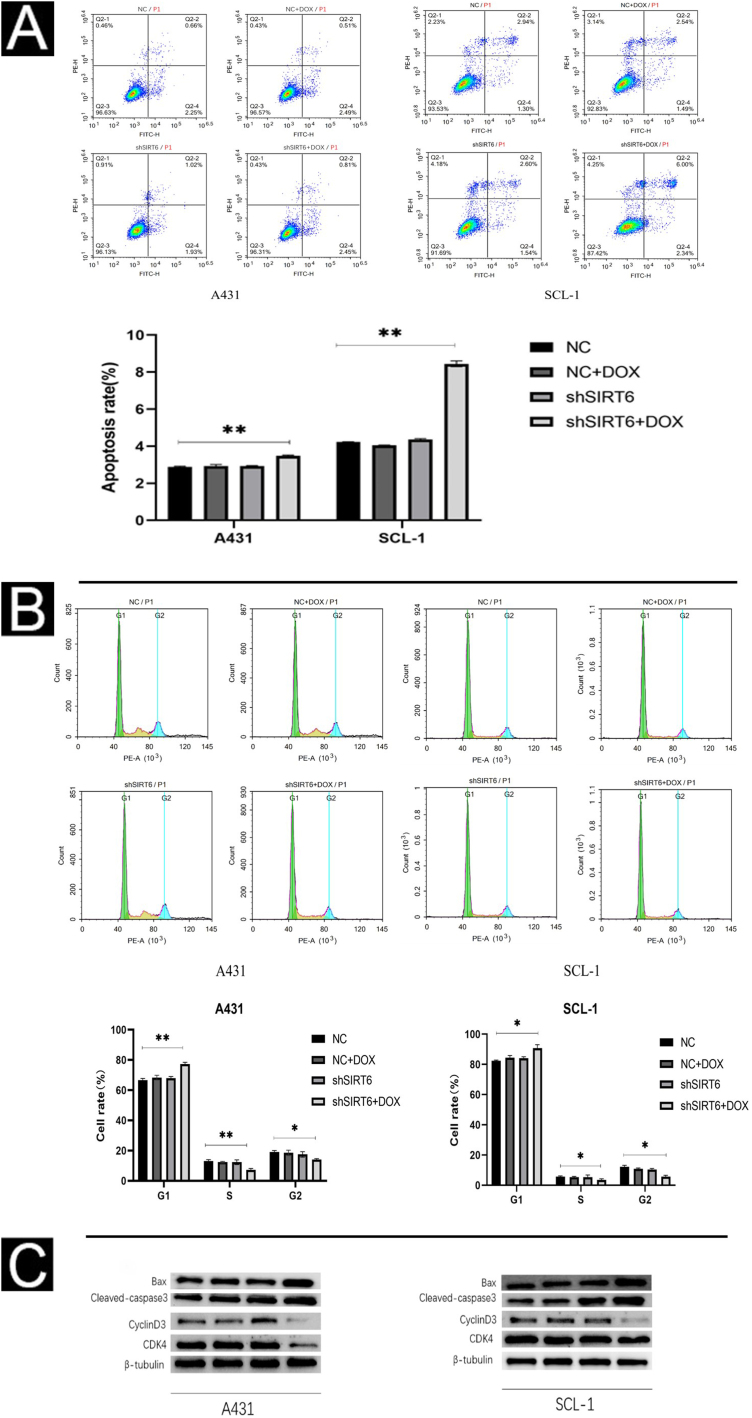
SIRT6 knockdown can increase apoptosis and cell cycle arrest in G0/G1 phase of CSCC cells. (A–B) Flow cytomatics analysis showed the apoptosis rate and cell cycle distribution of A431 and SCL-1 cells transfected with shSIRT6 + DOX and NC, NC + DOX and shSIRT6 in the control group. The apoptotic rate was measured by flow cytometry. The upper right quadrant represented late apoptotic cells, and the lower right quadrant represented early apoptotic cells. The apoptotic rate = late apoptotic ratio + early apoptotic ratio. (C) Western blots showed apoptosis-related proteins (Bax, Cleaved-caspase3) and cyclins (cyclinD1 and CDK4) in A431 and SCL-1 cells transfected with shSIRT6 + DOX and control NC, NC + DOX, shSIRT6 Flat, *p < 0.05, **p < 0.01.

Using flow cytometry, the authors further investigated the effect of SIRT6 on apoptosis and cell cycle progression in CSCC cells. The results show that the shSIRT6 + DOX-treated A431 and SCL-1 cell groups exhibit increased apoptotic rates (Fig. 4A) and blockage in the G0/G1 phase of the cell cycle (Fig. 4B), indicating that downregulation of SIRT6 can enhance apoptosis and induce cell cycle arrest at the G0/G1 phase in CSCC cells. To assess the expression of apoptosis-related markers (Bax, cleaved-caspase3) and cell cycle-related markers (CyclinD1, CDK4) in CSCC cells, Western blot analysis was performed. Compared to the NC, NC + DOX and shSIRT6 control groups, the shSIRT6 + DOX-treated A431 and SCL-1 cell groups exhibited increased levels of Bax and cleaved-caspase3 protein expression, while the levels of CyclinD1 and CDK4 protein expression were decreased (Fig. 4C). These results indicate that the downregulation of SIRT6 promotes apoptosis and induces cell cycle arrest at the G0/G1 phase in CSCC cells.

## Discussion

Cutaneous squamous cell carcinoma (CSCC) is a malignant proliferative tumor originating from the keratinizing cells of the appendages or epidermis. Although the majority of CSCCs can be successfully eradicated through surgical excision, approximately 14% to 15% of primary CSCC patients still experience subclinical infiltration.^22^ Invasive growth of CSCC can breach the basal membrane of the epidermis and invade the deeper dermis, skin appendages, blood vessels, lymphatic vessels, nerves, etc., leading to the development of high-risk CSCC. Despite significant advancements in treatment options such as Mohs surgery, cetuximab, and panitumumab, which have greatly improved the prognosis of CSCC patients, the prognosis for high-risk and very high-risk CSCC remains poor, with 5-year recurrence rates ranging from 30% to 50%.^23^ Therefore, it is necessary to further explore the mechanisms underlying the occurrence and progression of CSCC and identify more therapeutic targets.

SIRT6 is dysregulated in various cancers, and its role in tumorigenesis depends on the type and context of the tumor. SIRT6 expression is upregulated in breast cancer, pancreatic cancer, primary melanoma, and non-small cell lung cancer,^24^, ^25^ suggesting a pro-oncogenic role in these cancers. Conversely, SIRT6 is downregulated in ovarian cancer tissues, and its low expression is closely associated with tumor infiltration and metastasis.^26^ Studies have shown that SIRT6 inhibits the growth of gastric cancer by suppressing the JAK2/signal transducer and activator of the transcription-3 pathway.^27^ Furthermore, it has been reported that SIRT6 can suppress the occurrence and development of pancreatic cancer.^28^ Therefore, SIRT6 also plays a role in inhibiting the initiation and development of malignant tumors, with strong heterogeneity in different tumor types. The results of this study show that SIRT6 expression is increased in CSCC tumor tissues compared to normal skin tissues, suggesting that high expression of SIRT6 may be involved in the occurrence and development of CSCC. The protein expression level of SIRT6 in CSCC is higher than that in the precancerous lesion Actinic Keratosis (AK) and normal skin tissue, suggesting that SIRT6 may play an important role in the progression from normal skin tissue to precancerous lesions and further to Cutaneous Squamous Cell Carcinoma. However, SIRT6 expression was slightly higher in Bowen Disease (BD) than in CSCC tissue, which may be related to the limited number of BD tissue samples. High expression of SIRT6 in CSCC tissues is associated with the location of the cancer tissue in sun-exposed areas and Broders' grade, but not with gender, age, tumor diameter, etc., consistent with findings in melanoma and non-small cell lung cancer.^29^ Furthermore, the authors found that SIRT6 is highly expressed in CSCC cells and that silencing SIRT6 inhibits the proliferation, migration, and invasion abilities of CSCC cells. Invasion and migration are critical features of malignant tumors, and SIRT6 silencing significantly reduced the proliferation, migration, and invasion of CSCC cells, providing evidence for the role of SIRT6 in CSCC cell growth and motility.

Silencing of SIRT6 induces cell apoptosis and inhibits the G1/S cell cycle transition. Cell apoptosis is a complex process involving multiple signaling pathways, and the upregulation of apoptosis-related proteins (including Bax, cleaved-caspase3) following SIRT6 knockdown confirms the involvement of SIRT6 in the regulation of CSCC apoptosis. The authors also observed that SIRT6 knockdown leads to G0/G1 cell cycle arrest as demonstrated by cell cycle analysis. In addition, the downregulation of cyclin D1 and CDK4, which encode proteins that regulate the cell cycle, is associated with tumor progression.^30^ Cyclin D1 is a key protein in cell cycle regulation and is sufficient to drive cell cycle progression.^31^, ^32^ This study demonstrates the involvement of SIRT6 in the regulation of apoptosis and cell cycle in CSCC cells, thereby influencing the expression levels of apoptosis and cell cycle-related proteins. Based on this, the authors speculate that SIRT6 may be involved in the biological behavior of cutaneous squamous cell carcinoma cells as a functional molecule. However, there are several limitations to this study. First, the clinical tissue samples in this study are limited, and future studies should increase the sample size and use multiple experimental methods to further clarify the expression pattern of SIRT6. Secondly, due to the complexity of tumor regulation and development, the specific mechanisms are still subject to further investigation, and subsequent in vivo experiments should be conducted to explore the impact of SIRT6 on the biological behavior of CSCC. In addition, tumors are complex entities with high heterogeneity, and their occurrence and development are not determined by a single independent factor. Therefore, the identification of molecules in CSCC that may interact synergistically or antagonistically with SIRT6 remains a focus of future research.

## Conclusion

In summary, this study found that SIRT6 is highly expressed in CSCC, which may play a role as a carcinogenic factor, providing a certain theoretical basis for future treatment.

## Financial support

This work was supported by the project of Nantong Municipal Health Commission (MB2021019), the Basic Research Project of Nantong Science and Technology Bureau (JCZ2022021), and the Natural Science Foundation of Jiangsu Province (BK20211108).

## Authors’ contributions

Sai Chen: Study conception and planning; collection, analysis, and interpretation of data; statistical analysis; drafting and editing of the manuscript; critical review of the manuscript; approval of the final version of the manuscript.

Hongxia Chen: Collection, analysis, and interpretation of data; statistical analysis; drafting and editing of the manuscript; critical review of the manuscript; approval of the final version of the manuscript.

Xu Wang: Collection, analysis, and interpretation of data; statistical analysis; drafting and editing of the manuscript; critical review of the manuscript; approval of the final version of the manuscript.

Dongmei Zhang: Effective participation in research orientation; critical review of the literature; approval of the final version of the manuscript.

Li Zhang: Collection, analysis, and interpretation of data; critical review of the literature; approval of the final version of the manuscript.

Jiawei Cheng: Collection, analysis, and interpretation of data; critical review of the literature; approval of the final version of the manuscript.

Qi Zhang: Collection, analysis, and interpretation of data; critical review of the literature; approval of the final version of the manuscript.

Zhixiang Hua: Effective participation in research orientation; critical review of the literature; approval of the final version of the manuscript.

Xu Miao: Effective participation in research orientation; critical review of the literature; approval of the final version of the manuscript.

Jian Shi: Effective participation in research orientation; collection, analysis, and interpretation of data; statistical analysis; preparation and writing of the manuscript; critical review of the manuscript; approval of the final version of the manuscript.

## Conflicts of interest

None declared.
